# Prehabilitation for colon cancer surgery: a systematic review of quality-of-life outcomes and a feasibility study

**DOI:** 10.1007/s00384-026-05203-w

**Published:** 2026-07-25

**Authors:** Michelle V. Stadelhofer, Helene R. Dalby, Anne Thyø, Katrine J. Emmertsen

**Affiliations:** 1 Department of Abdominal Surgery, Regional Hospital Randers, Randers, Denmark; 2 Health Aarhus University, Ny Munkegade 120, Building 1521, 1st floor, DK-8000 Aarhus C, Denmark; 3 University Clinic for Coloproctology and Endoscopy, Regional Hospital Randers, Randers, Denmark

**Keywords:** Colorectal cancer, Prehabilitation, Feasibility, Systematic review, Quality of life (QoL), PROMS

## Abstract

**Purpose:**

To evaluate the feasibility of short-term prehabilitation and its impact on quality of life in patients with colorectal cancer through a systematic review and a feasibility study.

**Methods:**

The systematic review was conducted in accordance with the PRISMA guidelines. PubMed, PubPsych, CINAHL, EMBASE, Web of Science and EBSCOhost were searched for studies evaluating quality of life in colorectal cancer patients undergoing prehabilitation. We conducted a prospective non-randomised interventional feasibility study, with a 2-week post-surgery follow-up, enrolling patients diagnosed with colon cancer.

**Results:**

The systematic review included six studies from 517 unique records for final analysis. Among six studies, evidence for quality-of-life benefits of prehabilitation was conflicting but predominantly favourable, with generally low-to-moderate study quality and substantial heterogeneity. In the feasibility study, 110 patients were eligible for inclusion; 70 (64%) were included, and 63 completed the entire study. Of the 32 patients in the intervention group, compliance > 80% was observed in 66% (nutrition) and 88% (physical activity) of patients, with a median compliance of 100% for both. Quality-of-life outcomes were explored as secondary measures and showed potential favourable trends following prehabilitation.

**Conclusion:**

Evidence on the effect of prehabilitation on quality of life in colorectal cancer remains inconsistent; our feasibility study shows that short-term multimodal prehabilitation is feasible and supports the need for adequately powered trials.

**Supplementary Information:**

The online version contains supplementary material available at https://doi.org/10.1007/s00384-026-05203-w.

## 1. Introduction

Colorectal cancer is the third most common cancer worldwide [1], and surgical resection remains the only curative treatment [2]. Although advances in minimally invasive techniques have improved, short-term postoperative outcomes [3], particularly older and frail patients, can experience functional decline and reduced quality of life (QoL) following surgery [4–6]. Surgery triggers a cascade of stress-mediated responses in the body, including hypermetabolism, hypercatabolism and inflammation, which impair immune function and wound healing [7]. A prolonged disease course and postoperative complications are associated with an increased risk of worse long-term outcomes and poorer long-term QoL [8]. Prehabilitation can be a proactive approach to optimise the patients’ physical function, nutritional status and mental health before surgery, thereby improving post-operative recovery and outcomes [9–11]. Although prehabilitation may delay surgical treatment, it enables patients to remain actively engaged during the waiting period, fostering a greater sense of inclusion.

Despite increasing awareness and research on prehabilitation [10–12], substantial gaps remain regarding its effects on post-surgical recovery, QoL and compliance. This study aimed to evaluate prehabilitation for patients with colon cancer, with a specific focus on QoL.

The evaluation was performed in two ways: first, a systematic review was conducted to identify how QoL has been measured and reported in existing literature. Second, a feasibility study was conducted to evaluate the practicality and short-term outcomes of a multimodal prehabilitation programme within a clinical setting.

## 2. Methods

A systematic review was conducted to provide context, identify evidence gaps and support interpretation of the feasibility study findings. The systematic review and feasibility study were conducted during the same project period and were considered complementary components of this research, with the review situating the feasibility findings within the existing literature.

### 2.1 Method—systematic review

The study protocol was registered with Aarhus University, though not publicly available. The systematic review was conducted and reported according to the PRISMA guidelines [13, 14]. 

#### 2.1.1 Search strategy

The search strategy was developed in collaboration with a trained librarian. A comprehensive literature search was performed in the following databases: PubMed, PubPsych, CINAHL, EMBASE, Web of Science and EBSCOhost. Peer-reviewed studies evaluating the effect of prehabilitation on QoL in patients ≥ 18 years of age undergoing surgery for colorectal cancer were included. Case reports, case series, letters and editorials were excluded. No language restriction was employed. A translation system was applied if a study was available only in a foreign language.

The searches were performed on the 28th of August 2023 and updated on the 17th of October 2024, in accordance with the AMSTAR guidelines [15]. Searches span from the earliest available records to the search date. Supplement 1 elaborates on the search string and on the results from the first and second searches.

#### 2.1.2 Study selection and data extraction

After duplicate removal, titles, abstracts and full texts were screened by two independent authors (M.S. and A.T.) using the management software Covidence. Studies identified from conference abstracts or review citations were screened for full text. At all stages, disagreements were resolved by discussion. If consensus could not be reached, the last author (K.J.E.) made the final decision. The first author (M.S.) solely performed data extraction and quality assessment. A predefined customised data extraction sheet was used to extract data. All data on QoL outcomes were assessed.

#### 2.1.3 Quality assessment

For quality assessment, a tool specific to each study design was used in accordance with clinical guidelines. For randomised controlled trials (RCTs), the Cochrane-recommended risk-of-bias tool for randomised trials, version 2 (RoB2), was applied [16]. For cohort studies, the Newcastle-Ottawa scale (NOS) quality assessment form was used [17]. The RoB2 tool evaluates risk of bias across five main domains: (1) the randomisation process, (2) deviations from intended intervention, (3) missing data, (4) outcome measurement and (5) selection of results. Each domain was judged to have low risk, some concerns or high risk of bias, with an overall judgement derived from the domain-level assessment. The NOS comprises eight items distributed across three domains - Selection, Comparability and Outcome, each awarded stars for quality. To facilitate comparability across study designs, NOS scores of good, fair or poor quality were converted to the Agency for Healthcare Research and Quality (AHRQ) standards using previously established thresholds [18]. Studies were classified as good quality when they achieved high scores across all domains (≥ 3 in Selection, ≥ 1 in Comparability, ≥ 2 in Outcome). Fair quality corresponded to moderate scores (2/≥ 1/≥ 2), whereas poor quality reflected low performance in any domain (≤ 1/0/≤ 1).

#### 2.1.4 Data analysis

Due to heterogeneity in QoL instruments and inconsistency in the reporting of outcome domains, quantitative meta-analysis was not feasible. Therefore, a structured narrative synthesis with descriptive and comparative components was performed. Studies were compared based on overlapping QoL outcomes where possible, focusing on patterns in within-study changes over time and between-group differences. In cases where quantitative data were insufficient, findings were extracted from the written descriptions and conclusions.

### 2.2 Method—feasibility study

This was a non-randomised interventional feasibility study conducted in the Surgical Department at the Regional Hospital Randers, Denmark. All patients diagnosed with colon cancer were prospectively enrolled after being screened for eligibility from September 2022 to June 2023. Inclusion criteria were age ≥ 18 years and a macroscopically malignant tumour on endoscopy, later confirmed by pathological examination. Patients were excluded if they were ineligible for the intended curative colonic resection or unable to complete questionnaires due to language or cognitive impairment. Eligible patients were enrolled by a care coordinator on the day of cancer-suspect findings during colonoscopy or within 2 days by phone. The first author or a specialised care coordinator carried out enrolments. Consent was obtained from all participants after they received oral and written information about the study.

Patients diagnosed at Regional Hospital Randers were assigned to the intervention group, whereas patients referred to the hospital after a colonoscopy at another hospital or private clinic comprised the non-intervention group. All patients were approached and enrolled within three days of the diagnostic colonoscopy.

#### 2.2.1 Intervention and non-intervention groups

The short-term prehabilitation intervention consisted of a physical and nutritional component. The intervention group received education on the effects of nutrition and exercise on post-surgical recovery, a prescription for nutritional drinks and a kit containing a pamphlet with recipes for homemade supplements and samples of nutritional supplements. The patients were instructed to perform at least 20 min of unsupervised home-based moderate daily exercise (e.g. walking, aerobic training, resistance exercise or other preferred cardio activities) and to ingest three nutritional drinks (200 mL ∼ 300 kcal per drink) (20 g of protein) every day from study enrolment until surgery (approximately 2 weeks). Patients were advised to have a minimum protein intake of 80 g per day. If patients had conditions requiring special dietary attention (e.g. diabetes or kidney disease), a dietitian was consulted. The non-intervention group received standard care. In the Danish context, the standard care regimen for pre-surgical preparation includes information and advice on the effects of protein-rich nutrition and exercise until surgery, but without specific guidelines.

All patients underwent standard diagnostic evaluation, including CT imaging and histopathological confirmation of the cancer diagnosis, followed by an individual care discussion at a local multidisciplinary team meeting. Patients were subsequently seen in the surgical outpatient clinic for treatment planning and offered surgery within 10 days in accordance with Danish healthcare regulations.

#### 2.2.2 Outcomes and measures 

**Fig. 1:**
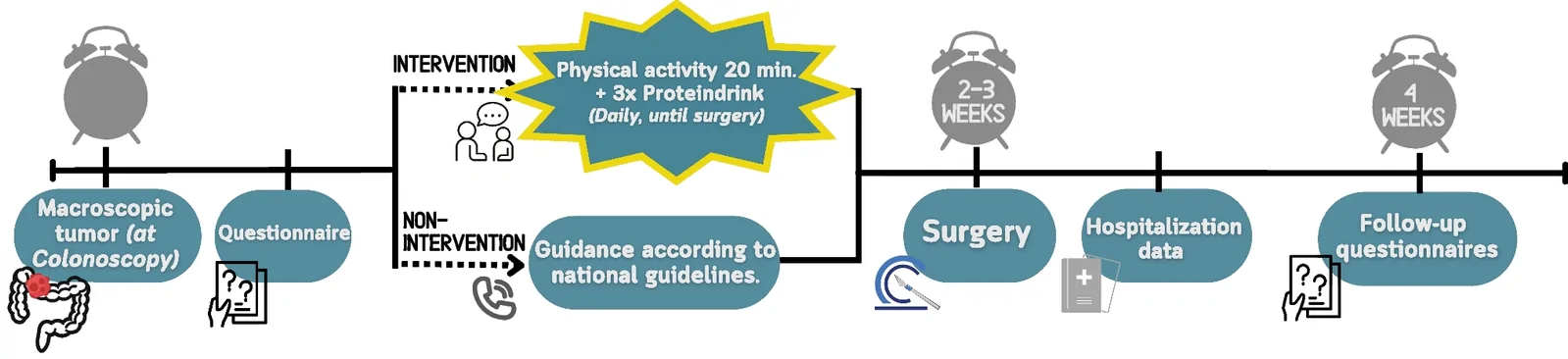
Study timeline and timepoints of data assessments

A timeline for outcome assessments is shown in Fig. 1. All patients completed a structured interview at inclusion and at follow-up, approximately two weeks post-surgery. The interview included patient-reported outcome measures (PROMs) assessing QoL and compliance with the intervention. The PROMs and additional questions were pilot tested in a focus group to ensure quality and comprehensibility. The final versions are available in Supplementary 2.

The primary outcome was intervention compliance, assessed through patient self-reporting at follow-up. Participants recorded the number of days they adhered to the prescribed intervention. Compliance was defined as the extent to which patients adhered to the intervention instructions.

A percentage score was subsequently calculated as the proportion of nutritional drinks consumed: $\frac{Number\;of\;consumed\;nutritional\;drinks}{Optimal\;number\;of\;nutritional\;drinks}\ast100\%$. The same methodology was applied to assess physical activity. There is currently no validated gold standard for defining an acceptable adherence threshold in prehabilitation research. Historically, an arbitrary rate of 80% or higher has been considered good compliance [19, 20].

Secondary outcomes included both clinical and patient-reported measures (PROMS). Clinical data extracted from medical records comprised weight change during the study period, length of hospital stay (LOS) and surgical and medical complications. Complications were categorised by severity using the Clavien-Dindo classification system, ranging from grade 0 (no complication) to grade 5 (death) [21]. Weight was measured at baseline and on the day of surgery.

PROMs included ad hoc questions exploring patients’ sense of inclusion within the disease course and self-reported cancer-related symptoms, as well as validated instruments assessing quality of recovery (QoR) by Quality of Recovery-15 (QOR-15) [22] and QoL using the EuroQol (EQ)-5D-5L and EQ visual analogue scale (VAS) [23, 24]. All instruments have previously been validated in a Danish cohort [24–26]. The QOR-15 encompasses 15 domains related to post-surgery daily activities and symptoms. Scores range from 0 to 150, with higher scores indicating better post-surgery recovery. The EuroQol (EQ)−-5D-5L [23, 24] evaluates five domains (mobility, personal hygiene, usual activities, pain and anxiety/depression) on five levels, reflecting the patients’ perception of their health status, while the VAS score evaluated the patients’ self-rated health on a visual scale ranging from 0 to 100, where 100 is labelled “the best health you can imagine” and 0 “the worst health you can imagine”. The EQ-5D-5L and VAS scores were measured at both baseline and follow-up.

Evaluation of patients’ feelings of inclusion was incorporated in the post-surgery questionnaire. Patients were asked to what degree they felt they had a role in their disease course. Five degrees were presented, ranging from "not at all" to "a very high degree". Self-reported cancer symptoms were assessed at baseline. Patients were asked about unintentional weight loss in the previous six months and reduced appetite or fatigue in the past two weeks.

#### 2.2.3 Follow-up assessment

Follow-up was scheduled at the patients’ routine clinic appointment 13–15 days post-surgery, when they were seen for a postoperative check-up and received their final histopathology results of the colon resection (Fig. 1). The first author administered the follow-up questionnaire immediately before the appointment with the surgeon to minimise bias related to prognosis and subsequent treatment decisions.

#### 2.2.4 Statistical analysis

Data analyses were performed in R (2023). Descriptive statistics were compiled to characterise the study population, with quantitative data summarised as medians with interquartile range (IQR), and categorical variables summarised as counts and percentages. As a feasibility study, data analysis was primarily descriptive and exploratory. Group differences in categorical variables were assessed using Fisher’s exact test or Pearson’s chi-squared test, as appropriate. Continuous variables were compared using *t*-tests for parametric data and the Wilcoxon rank-sum test for non-parametric data. All analyses were exploratory, as the study was not powered to detect statistically significant differences. Any statistically significant findings should therefore be interpreted with caution, as highlighted in the discussion session.

## 3. Results

### 3.1 Results—systematic review

**Fig. 2:**
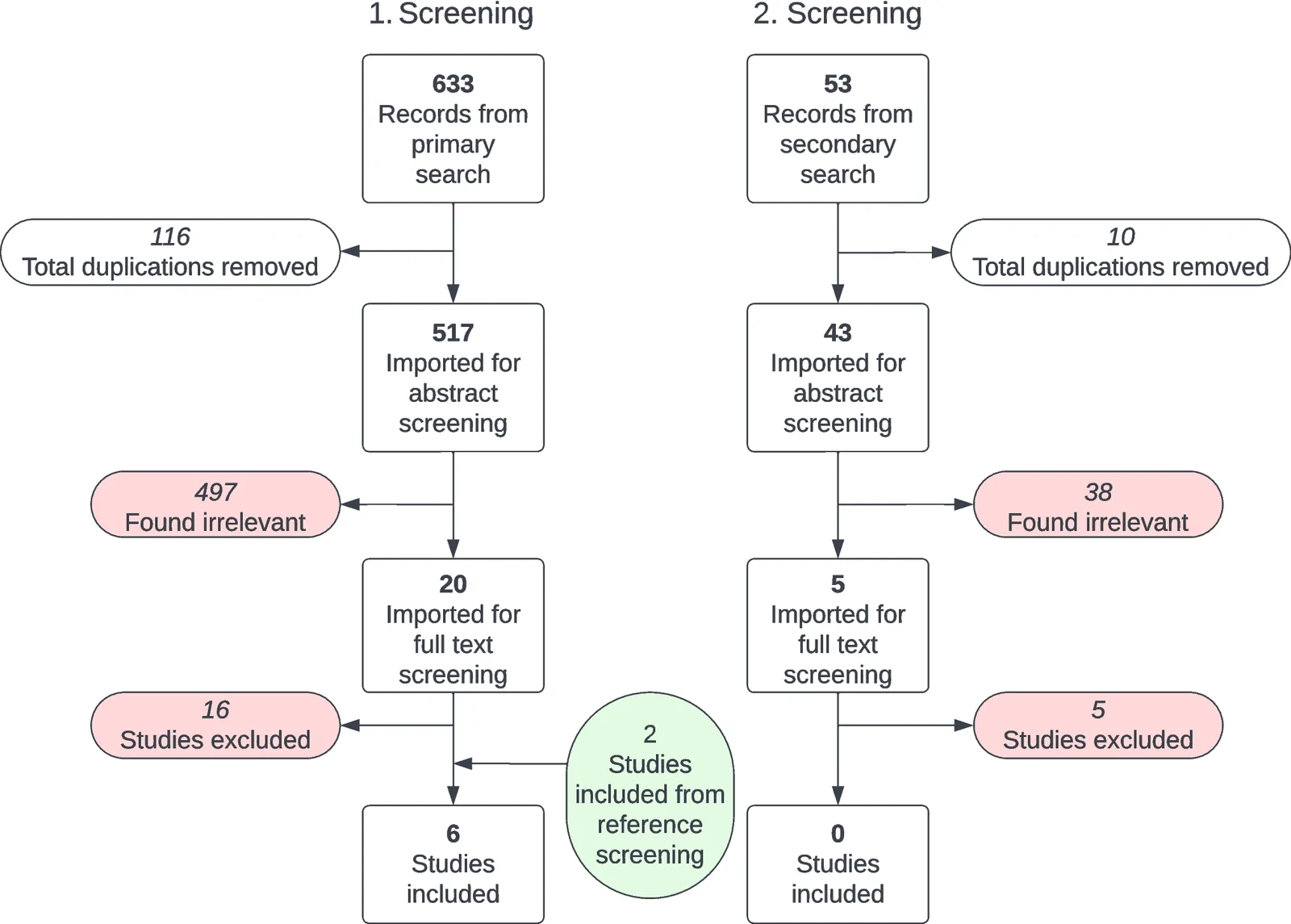
PRISMA flowchart of the screening process

The primary literature search identified 633 records. After removing duplicates, 517 unique records were screened. The PRISMA flowchart is illustrated in Fig. 2. Studies were primarily excluded because QoL was not measured. A total of six studies were included in the final analysis [27–32]. The updated literature search yielded no records for inclusion. Study characteristics are presented in Table 1, including the number of participants included, the composition of the interventions and the quality assessment. Only the cohort study by Li et al. [31] was assessed as good quality. Two studies [30, 32] received an overall assessment of poor quality, primarily due to a high number of participants lost to follow-up or missing data. The remaining three studies were evaluated as of moderate/fair quality [27–29].

**Table 1:**
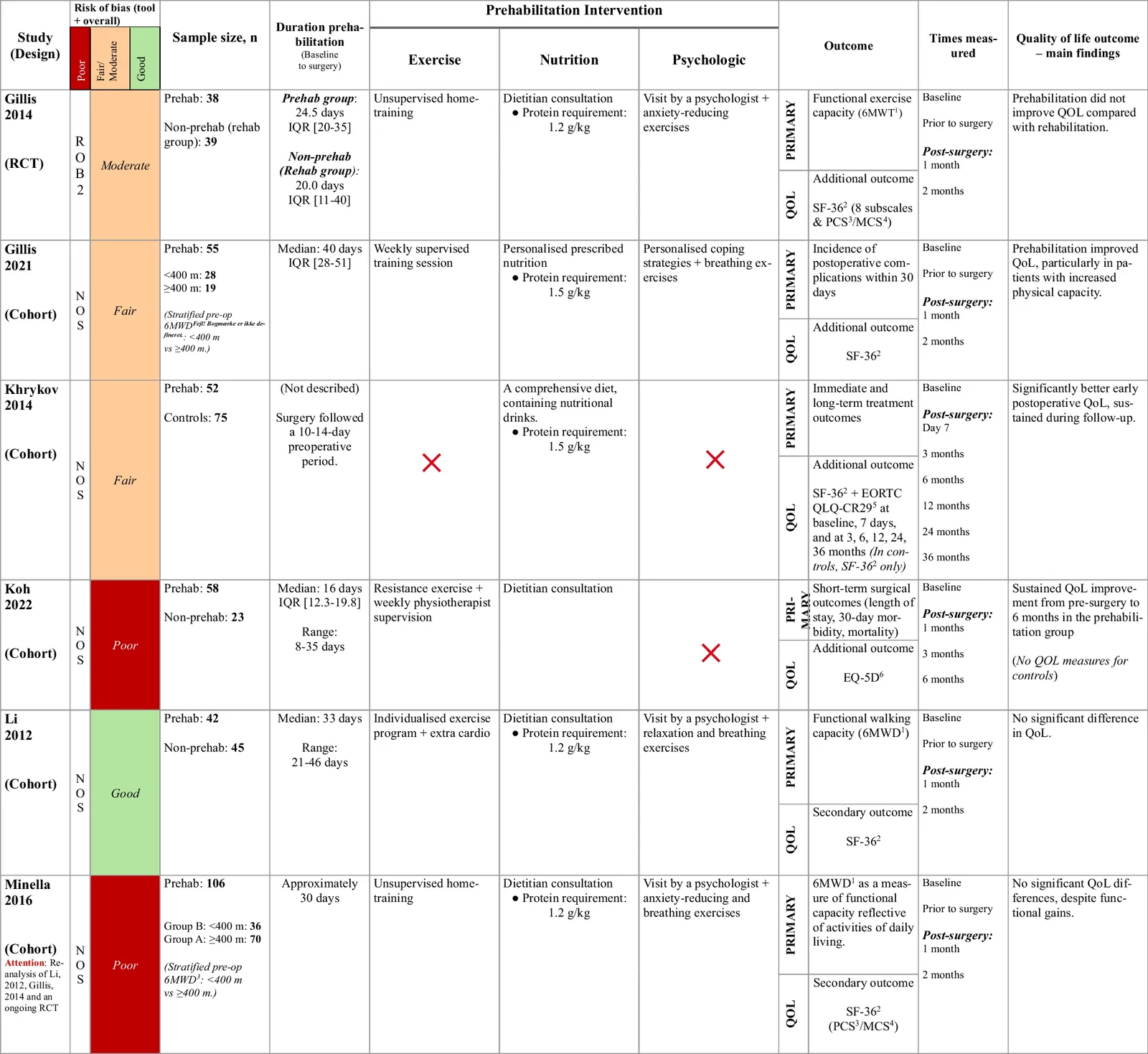
Overview of the final included studies in the systematic review

^1^6MWD = 6-minute walk test (measures the distance that a patient can briskly walk on a flat, hard surface over a period of 6 minutes)

^2^SF-36 = Short Form‑36 Health Survey

^3^PCS = Physical Component Scale (SF-36 component)

^4^MCS = Mental Component Scale (SF-36 component)

^5^EORTC QLQ-CR29 = European Organisation for Research and Treatment of Cancer – Quality of Life Questionnaire, Colorectal Cancer Module

^6^EQ-5D = European Quality of Life - 5 Dimensions

#### 3.1.1 Characteristics of the prehabilitation programmes

The included studies varied substantially in their prehabilitation interventions with respect to modality (uni-, multi-, or trimodal), duration and specific intervention characteristics, such as the intensity of physical activity (Table 1). Four studies [27, 29–31] analysed QoL scores comparing a rehabilitation group with a control group. In general, the studies reported high levels of compliance with the prehabilitation programme.

#### 3.1.2 Quality of life outcomes

None of the included studies measured QoL as a primary outcome; it was measured only as a secondary or additional outcome. The studies primarily employed the 36-Item Short-Form Health Survey (SF-36). Table 1 provides further details on outcome measures.

Due to heterogeneity across studies, particularly in the intervention programmes, a meta-analysis could not be performed. Instead, studies have been systematically compared. A detailed illustration of the available data for QoL outcomes is shown in Table 2.. Four studies [28–30, 32] reported that prehabilitation had a positive effect on QoL, while two studies [27, 31] found no improvement. One study [29] reported a statistically significant improvement in the prehabilitation group compared to the control group at seven days post-surgery, whereas two studies [27, 31] found no significant differences between the prehabilitation and comparison groups.

**Table 2.**
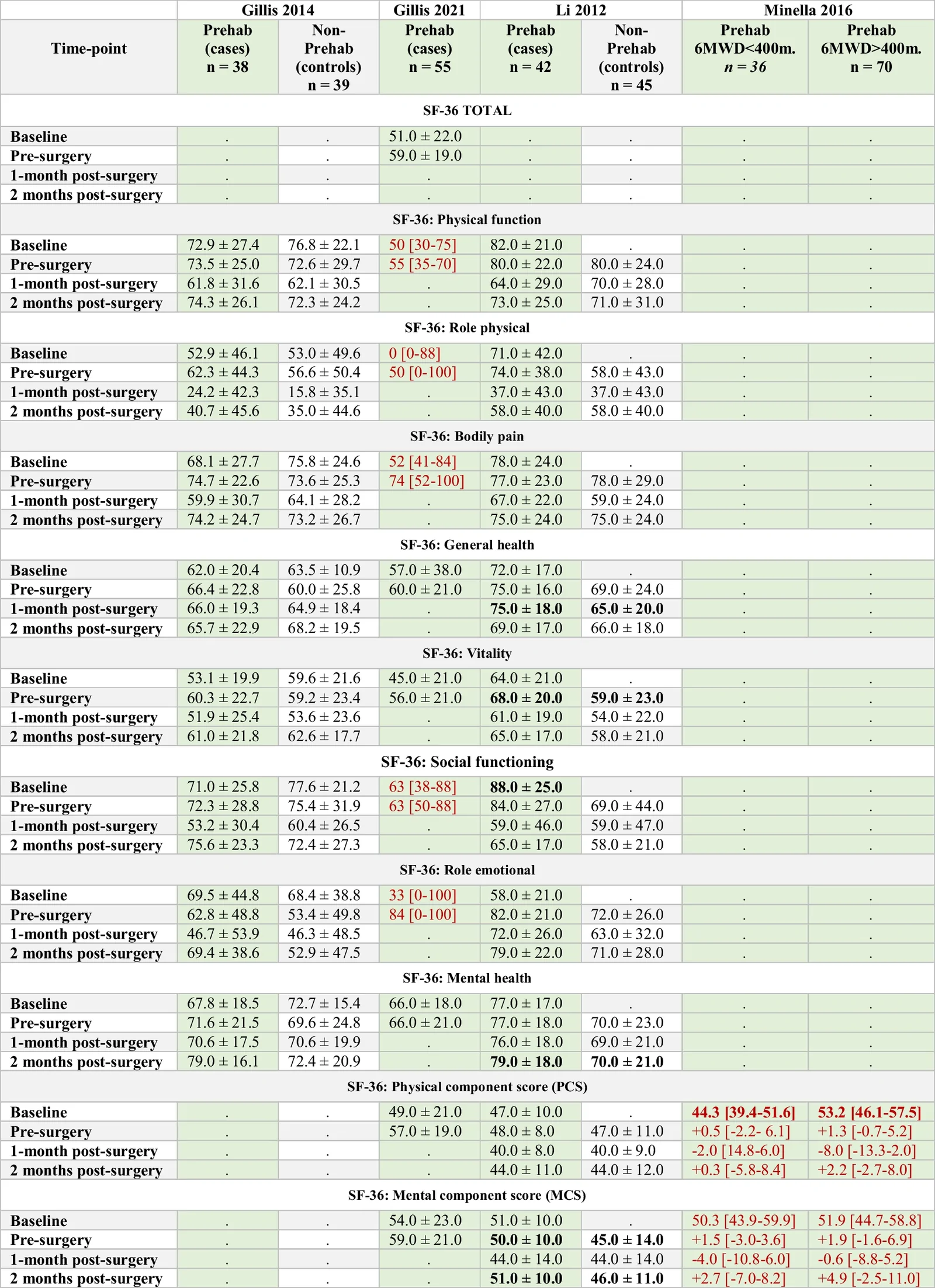
QoL outcomes using SF-36

mean values ±SD. The green columns indicate results for patients in a prehabilitation group. Red text = median values [IQR]. Bold text indicates that results were statistically significant

### 3.2 Results—feasibility study

**Fig. 3:**
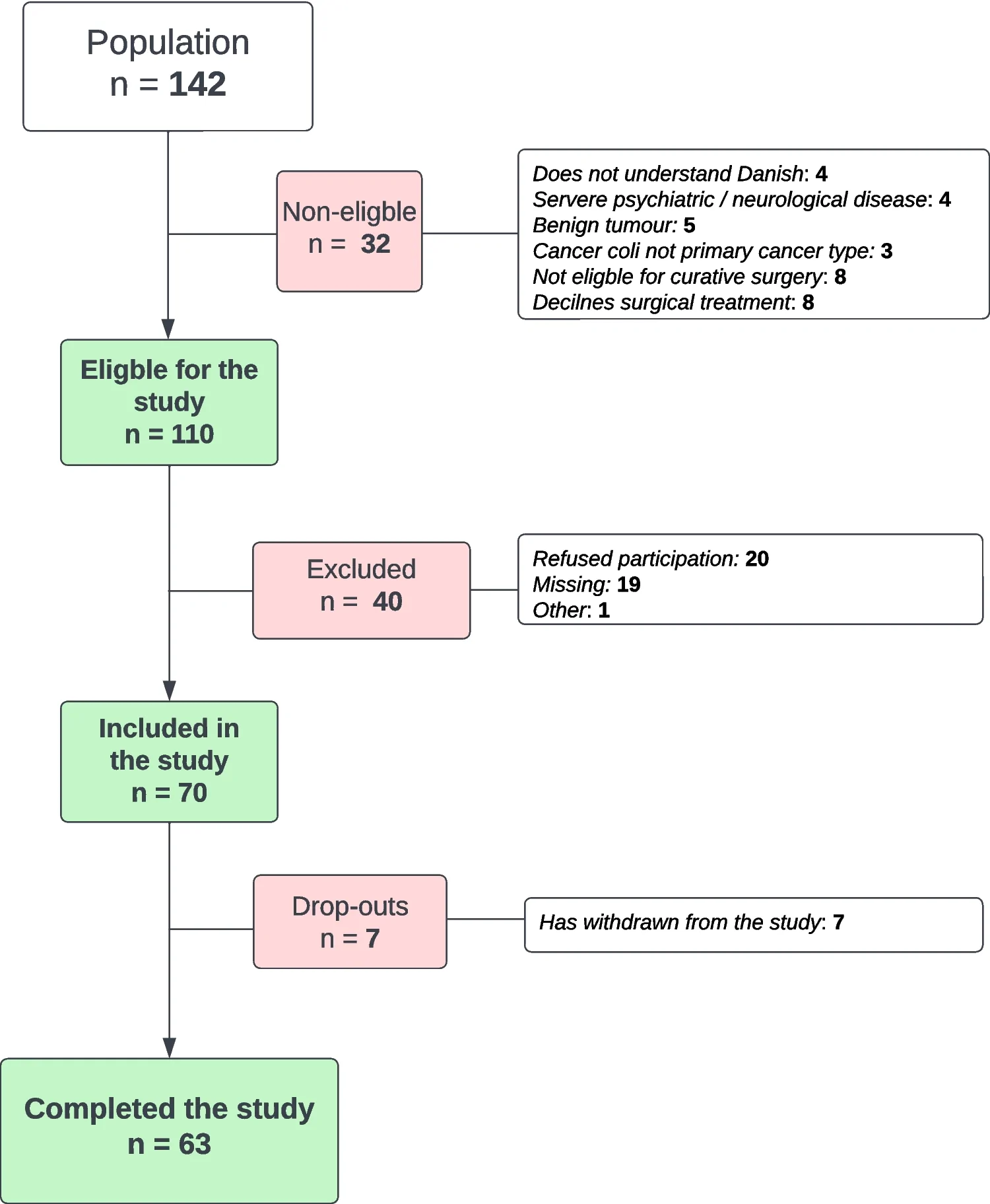
Participant flow and data completeness. Missing participants mainly refer to cases where contact could not be established or where patients were not offered the opportunity to participate

Of the 110 eligible patients, 70 (64%) were included in the study. Of these, 63 patients (90%) completed the follow-up (Fig. 3). No significant differences in age or gender were observed between included and excluded patients. Similarly, analysis of dropouts and patients excluded after baseline revealed no differences in baseline characteristics. Baseline characteristics and operative characteristics are shown in Table 3. Overall, no significant differences in baseline characteristics were observed between the two groups. However, the non-intervention group had a higher proportion of patients with indicators of frailty (higher ASA and PS scores). The interval between the initial endoscopy and the surgery date was 2 to 3 weeks.

**Table 3:** Baseline, surgery and follow-up characteristics

Characteristic	No. (%)	
	Intervention group (*n* = 32)	Non-intervention group (*n* = 31)
**Age**, median (IQR), years	69.5 (64.8, 74.5)	75.0 (66.5, 80.0)
**Sex**		
Female	19 (59%)	15 (48%)
Male	13 (41%)	16 (52%)
**BMI**, median (IQR)	25.8 (24.3–29.3)	25.2 (22.6–28.2)
**ASA group**		
ASA I-2	28 (88%)	21 (68%)
ASA 3–4	4 (13%)	10 (32%)
**Performance status (PS)**^**1**^		
PS 0	27 (84%)	20 (65%)
PS 1–3	5 (16%)	11 (35%)
**Smoking status**		
Active smoker	3 (9%)	1 (3%)
Previous smoker^2^	15 (47%)	17 (55%)
Never smoked	14 (44%)	13 (42%)
**Weekly alcohol**		
< 10 units weekly	25 (78%)	25 (81%)
≥ 10 units weekly	7 (22%)	6 (19%)
**Tumour stage (UICC-stage)**		
I/II	20 (62.5%)	21 (67.7%)
III	12 (37.5%)	8 (25.8%)
IV	0 (0%)	2 (6.5%)
**Days until surgery**, median (IQR)	14.0 (13–16)	18.0 (15–20)
Surgery and follow-up		
	Intervention group (*n* = 32)	Non-intervention group (*n* = 31)
**Type of surgery**		
Hemicolectomy dxt	22 (68.8%)	23 (74.2%)
Hemicolectomy sin *or* sigmoidectomy	10 (31.2%)	8 (25.8%)
**Surgical technique**		
Laparoscopic	27 (84%)	29 (94%)
Primary laparoscopic converted to open	5 (16%)	2 (6.5%)
**Primary anastomosis or stoma**		
Primary anastomosis	30 (94%)	30 (97%)
Stoma	2 (6%)	1 (3%)
**Length of stay (LOS)**, days, median (IQR)	3.5 (3.0–8.3)	4.0 (3.0–5.0)
**Length of stay (LOS)**, days, mean (range)	6.2 (3.0–19.0)	5.6 (2.0–23.0)

^1^Performance status scores were graded from 0 to 4: 0 indicates fully active without physical restrictions and 4 indicates need for round-the-clock care

^2^No smoking in the previous 8 weeks

#### 3.2.1 Compliance with the prehabilitation intervention

After inclusion, seven (10%) patients withdrew from the study. Among patients who completed the study, the mean compliance score for the nutritional intervention was 80% (IQR 65-100), and for the physical activity intervention, 89% (IQR 71-100). A nutritional component compliance score exceeding 80% was achieved by 66% of all patients, with a median compliance value of 100%. Compliance was slightly higher for the physical activity component, with 88% of all patients scoring above 80%, and a median compliance of 100%.

#### 3.2.2 Feeling of inclusion in one’s own course of the disease

In the intervention group, 38% of patients reported not feeling included, compared with 70% in the non-intervention group. In the intervention group, the majority reported feeling included to a very high (9%), high (16%) or moderate degree (28%), whereas only a minority reported feeling included to a low degree (9%). In contrast, the non-intervention group showed a markedly different distribution, with only one (3%) reporting a very high degree of feeling included, and fewer patients indicating high (10%), moderate (10%) and low (7%) degrees of inclusion.

#### 3.2.3 Quality of life—the EQ-5D-5L

**Fig. 4:**
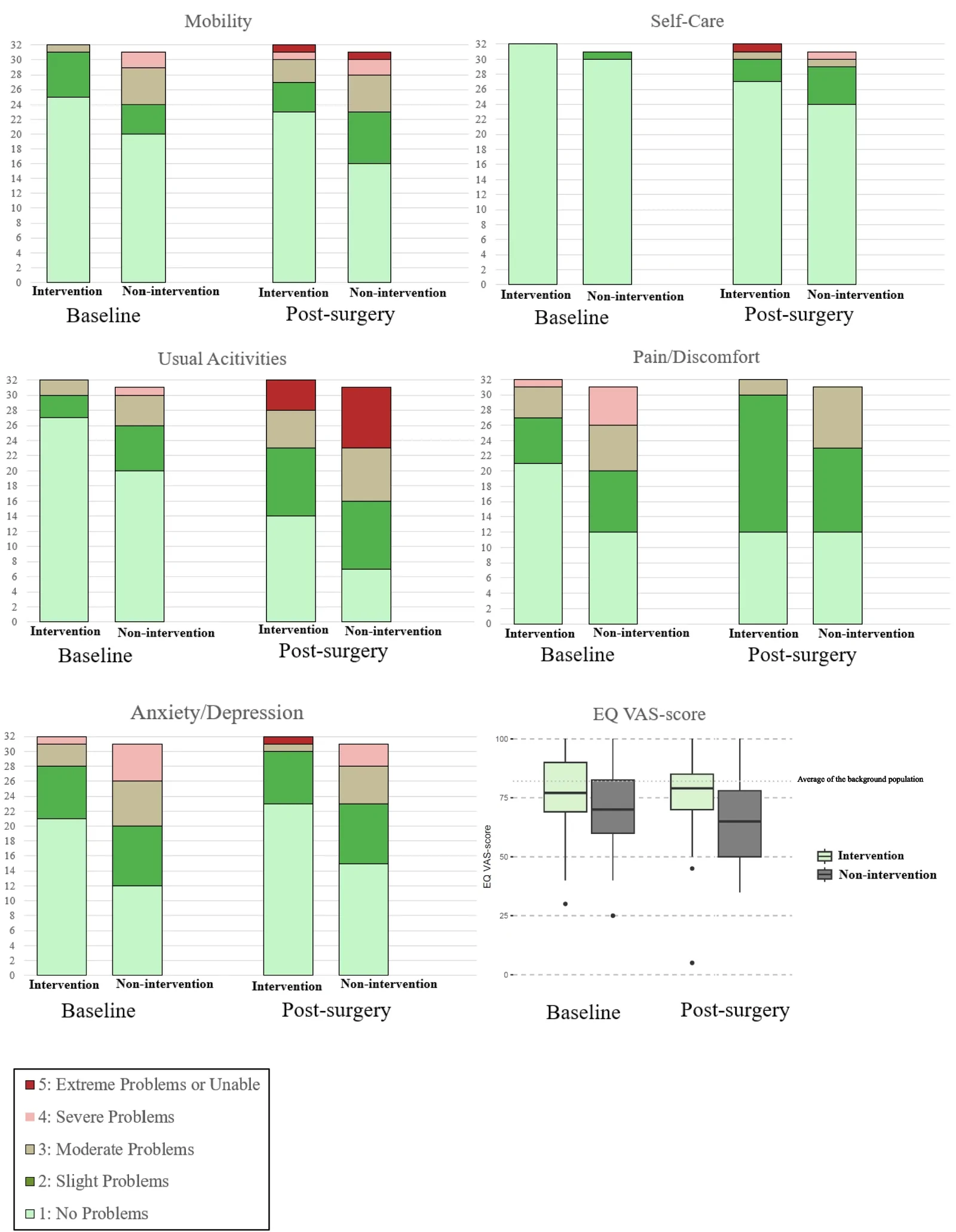
EQ-5D-5L at baseline and post-surgery for the intervention and the non-intervention group. Including a box plot of the VAS score of self-reported general health

Figure 4 shows the distribution of EQ-5D-5L responses at baseline and follow-up (post-surgery) for the intervention and non-intervention groups. At baseline, domain profiles were broadly comparable. Except for anxiety and depression, both groups demonstrated a decline across all severity types from baseline to the post-surgical period.

Post-surgery, several notable trends emerged. For usual activities, the non-intervention group demonstrated a marked shift towards moderate-to-severe problems. In contrast, the intervention group retained a higher proportion in the no or only slight problems categories. As for anxiety/depression, the intervention group generally reported a more favourable distribution with a lesser degree of problems compared to the non-intervention group. The intervention group also reported fewer moderate-severe problems than the non-intervention group. The proportion of patients reporting pain/discomfort was comparable post-surgery, although patients in the non-intervention group reported pain/discomfort more frequently at baseline. 

In an exploratory analysis post-surgery, EQ-VAS scores were higher in the intervention group than in the non-intervention group (*p* = 0.047). Scores increased slightly from baseline to post-surgery in the intervention group (from a median of 77.0 to 79.0), whereas they declined in the non-intervention group (from a median of 70.0 to 65.0).

#### 3.2.4 Additional outcomes

At baseline, patients exhibited a wide range of cancer-related symptoms. Approximately 33% had experienced unintended moderate weight loss and reported a reduced appetite compared to normal. More than half reported some degree of fatigue, and 25% presented with anaemia at diagnosis.

From baseline to surgery, weight change was small overall, with a median gain of 0.45 kg (IQR −0.70 to 1.53). Most patients maintained a relatively stable weight during the preoperative period and did not experience further weight loss. Weight data on the day of surgery were missing for 15 patients (9 in the intervention group and 6 in the non-intervention group). For these patients, weight was obtained on the first or second postoperative day. Only five patients had no post-surgery weight data available. 

During hospitalisation, patients in the two groups showed similar patterns. Patients in the intervention group had a median LOS of 3.5 days (IQR 3.0-8.3), and those in the non-intervention group had a median LOS of 4.0 days (IQR 3.0-5.0). Generally, the number of perioperative and post-surgical complications was low and evenly distributed between the two groups, as was the case when converted to the Clavien-Dindo classification. The most common post-surgery complications were paralytic ileus, incisional hernia and anastomotic leakages, with three patients experiencing each outcome. Anastomotic leakage was observed in two patients in the intervention group and in one in the non-intervention group.

The QoR scores were similar between groups, with similar medians and overlapping interquartile ranges. The non-intervention group exhibited a wider score distribution and more low-value outliers, whereas the intervention group showed a more clustered score profile (data not shown).

## 4. Discussion

The systematic review updates the evidence of the effect of prehabilitation on QoL in patients undergoing surgery for colorectal cancer. Although the included studies reported heterogeneous findings, most suggested potential improvements in QoL associated with prehabilitation, with only one demonstrating a statistically significant benefit compared to controls. 

The feasibility study primarily evaluated compliance with a short-term multimodal prehabilitation programme in patients undergoing surgery for colonic cancer. Overall compliance was high, with 66% of participants achieving > 80% compliance with the nutritional component and 88% with the physical activity component. This demonstrates that prehabilitation is feasible in this clinical setting without compromising the planned surgical treatment.

Since this clinical trial was designed as a feasibility study, the secondary outcomes should be interpreted with caution, given its exploratory nature, small sample size and the fact that the study was not powered to assess causal effects. Our findings should not be interpreted as evidence of prehabilitation effects but should be considered hypothesis-generating. Nevertheless, they provide insights into potential patterns. Consistent with the systematic review, QoL outcomes suggested possible favourable trends following prehabilitation, particularly in domains of returning to usual activities and reducing anxiety and depression, although none of these differences reached statistical significance. Self-perceived general health scores (EQ-VAS scores) were higher in the intervention group at 2 weeks post-surgery. However, this exploratory finding should be interpreted cautiously and requires confirmation in a larger, adequately powered trial.

Our findings for global QoL are consistent with prior cancer-specific trials and reviews that have reported mixed effects on global QoL scores. Earlier studies [12, 33, 34] more often reported no change or a decline in QoL scores, whereas more recent studies consistently report a relatively small but positive effect of prehabilitation on QoL or differences between groups [35, 36]. A systematic review [37] and a Cochrane review [38] focusing exclusively on colorectal cancer have recently been published. However, as these reviews included only RCTs, they were unable to analyse QoL outcomes due to the limited number of eligible studies. To our knowledge, the present review is the first to specifically focus on prehabilitation for colorectal cancer and QoL outcomes across both RCTs and observational studies. The inclusion of diverse study designs inevitably introduced heterogeneity among the included studies, further compounded by variation in prehabilitation modalities, intervention duration and outcome measures. Similar challenges have been reported in other reviews in this field, including that by Chou et al. [39].

Our systematic review found that only one study [31] had a low risk of bias, while the rest were rated as fair to poor quality. A key methodological limitation across several studies was the small number of patients undergoing prehabilitation. In addition, the assessment of QoL represented a recurring methodological concern, reflecting heterogeneity in measurement instruments and reporting. Despite the use of validated questionnaires across studies, the assessment of QoL remains subjective and influenced by multiple perioperative and psychosocial factors. Furthermore, scores may be influenced by recall or timing-related bias. The generally low methodological quality of studies evaluating QoL outcomes could partly reflect that QoL is often considered a secondary endpoint and assessed over relatively short follow-up periods. In addition, differences in outcome definitions, subdomains and measurement instruments limit comparability across and reduce the feasibility of pooled analyses. These limitations highlight the need for greater standardisation of QoL assessment in colorectal prehabilitation research, including predefined assessment time points and, where appropriate, the evaluation of QoL as a primary endpoint.

International variation in prehabilitation practices poses challenges for standardising. Programmes differ in duration, modality, target population and intervention intensity, complicating comparisons between interventions and their effects. The optimal timing for colorectal cancer surgery has been discussed in the literature, with suggestions ranging from 2 to 6 weeks [40]. In Denmark, the maximum waiting period from final diagnosis of colorectal cancer to surgery is 10 days. However, under recent Danish legislation, surgeons are allowed to postpone surgery to prescribe prehabilitation if deemed necessary to optimise the patient before surgery [41]. The feasibility study provides valuable insight, showing that even a brief prehabilitation intervention can achieve high compliance, particularly for the nutritional and physical activity components. This is clinically relevant, as the limited time between diagnosis and surgery is frequently cited as a key barrier to implementing prehabilitation [42].

Compared with the existing literature, several prehabilitation studies across various cancer types have demonstrated similar compliance rates of 70-80% or higher [11, 43, 44]. This trend was also observed in several multimodal studies included in our systematic review [27, 28, 31]. Consistent with our findings, several studies have found that compliance with physical activity interventions tends to be higher than with nutritional interventions. The very high compliance observed with the physical activity intervention is likely due to its simple, flexible nature, which allows for individual patient interpretation. From a clinical perspective, these combined findings support the feasibility of incorporating a short-term multimodal prehabilitation into colorectal cancer treatment pathways. This perspective is consistent with recent developments in colorectal cancer care, including contemporary evidence from minimally invasive surgery demonstrating favourable long-term oncological outcomes [45]. Although such studies primarily focus on surgical and oncological outcomes, they also reflect a broader shift in colorectal cancer care, in which technical surgical success is increasingly complemented by perioperative optimisation, recovery pathways and patient-centred supportive strategies. 

We acknowledge that in a trial setting, additional resources may be available, which may contribute to higher patient compliance. Nevertheless, the observed trends may indicate greater patient involvement in their disease course. These findings should be interpreted with caution and as exploratory, suggesting that future adequately powered studies should examine whether short-term prehabilitation may promote higher compliance and potential benefits by enhancing patient engagement, empowerment and preparedness.

### 4.1 Strengths and limitations

The main strength of this work is the integration of a systematic synthesis of evidence with original feasibility data, enabling combined interpretations. The systematic review followed PRISMA guidelines, and searches were developed in collaboration with a librarian and employed a clearly defined search strategy across a comprehensive range of databases without language restrictions. Reference lists were screened, and appropriate quality assessment tools were carefully selected for each specific study design.

A key strength of the feasibility study is the low drop-out rate (10%). Patients who withdrew did not differ significantly from those who completed the study in terms of age, gender, ASA classification, performance status or BMI. Another notable strength is the inclusion of patient-centred outcomes that go beyond traditional QoL measures, such as feelings of inclusion and QoR, assessed within a non-randomised controlled design. Although patient involvement in care is increasingly recognised as a crucial factor for satisfaction and perceived benefit, it remains underreported in prehabilitation research.

There are some limitations to our studies. Within the systematic review, the broad inclusion of study designs inevitably introduced heterogeneity among the included studies. Although the search was updated in 2024, publication of our findings was delayed by over a year, and no additional updates were made. Data extraction and quality assessment were performed by a single reviewer, which may have introduced reviewer bias.

The feasibility study was non-randomised, with group allocation determined by referral pathways rather than by random assignment. This introduced a substantial risk of selection bias. The small sample size represents an additional limitation, and together with its design, the study was not powered to assess efficacy outcomes, limiting causal inference drawing and reducing generalisability. Although the inclusion rate was 64%, legal constraints prevented data collection from non-participants, thereby preventing statistical comparisons. Importantly, at baseline, the non-intervention group appeared frailer (higher proportions of ASA 3-4 and PS 1-3), which may have influenced QoL and postoperative recovery outcomes independently of prehabilitation. Prehabilitation is considered particularly beneficial for frail patients [46]. However, such patients are challenging to involve in research. It is possible that some very frail patients were not included in the study, potentially introducing selection bias and affecting internal validity. Finally, compliance scores and PROMS were primarily based on self-reported data and may be subject to recall bias.

### 4.2 Future aspects

While our prehabilitation intervention is feasible to implement, the future application of prehabilitation on a larger scale faces several challenges, particularly in logistics and resource allocation within a hospital’s daily workflow. Previous research in this field suggests that applying a multimodal or trimodal prehabilitation lasting for at least 3 weeks is necessary to achieve full benefits [46]. To achieve greater consistency, there is a need for consensus on the definitions of prehabilitation, intervention modalities and the screening tools employed to determine when to implement a prehabilitation programme. A recent Delphi study by Fish et al. [47] proposed a more standardised approach to prehabilitation and may provide guidance for future trials. Greater standardisation could lead to higher-quality, more homogeneous evidence, facilitating synthesis and improving the translation of prehabilitation research into patient benefit. Obstacles also include the lack of qualified staff and restricted financial resources. Moreover, there is a need for studies evaluating the economic impact of prehabilitation in this patient population, even though early prehabilitation interventions may be cost-effective by reducing the burden of postoperative complications.

To evaluate the causal effects of prehabilitation on outcomes such as QoL, post-surgery complications, QoR and patients’ sense of inclusion, more high-quality RCTs with large sample sizes are needed. Although satisfactory compliance was observed, the effectiveness of short-term prehabilitation should be evaluated in longer and more comprehensive programmes. In addition, clearer guidelines are needed on when curative surgery should be postponed in favour of prehabilitation and how best to identify patients most likely to benefit.

## 5. Conclusion

Overall, our findings suggest that, while robust evidence for definitive effects of prehabilitation on QoL remains limited, prehabilitation appears acceptable, implementable and potentially beneficial from a patient perspective. This supports further evaluation in adequately powered trials with QoL as a primary outcome. The studies included in the review showed substantial heterogeneity and, at times, conflicting results, underscoring the need for additional high-quality research. Our feasibility study further suggested potential favourable trends across PROMs, including QoL-related domains and patients’ perceived feelings of inclusion. However, given the exploratory nature of this study, these findings should be interpreted with caution and considered hypothesis-generating. Future research should aim to establish consensus-based intervention frameworks, incorporate economic cost-benefit analyses, develop guidance on optimal timing, duration and patient selection, and work towards greater standardisation of QoL assessment in colorectal prehabilitation studies.

## Supplementary Information

Below is the link to the electronic supplementary material.

ESM1 (PDF 176 KB)

ESM2 (PDF 353 KB)

## Data Availability

The data supporting the findings of this study are available within the article and its Supplementary Information. The questionnaires used or created are available in supplementary 2. Any additional data might be available from the corresponding author upon reasonable request. These data have not been made publicly available because they involve human research participants.
