# Supplementary material for: Prehabilitation for colon cancer surgery: a systematic review of quality-of-life outcomes and a feasibility study
Source: Int J Colorectal Dis. 2026 Jul 25;41(1):165. doi: 10.1007/s00384-026-05203-w (PMC13683530; doi:10.1007/s00384-026-05203-w)
Supplement: Supplementary file 1 [file 384_2026_5203_MOESM1_ESM.pdf]

Appendix 1

Appendix 1 – Search string and details of the searches, including number of studies

**Search string**	(Prehabilitation* OR optimi* OR intervention* OR nutrion* (OR "Nutrional optimization") OR excercise* OR physical activity* OR psychology OR psychological OR smoking)<br>AND (major surgery OR colon resection)<br>AND (Colon OR rectum OR colorect*)<br>AND (Cancer OR Neoplasm)<br>AND ("Quality of life" OR quality OR patient-reported outcome OR PRO OR PROM)<br><br>***MESH-terms, index terms or similar specialized search methods were applied and modified for each database.***

	**Primary search** (28.08.2023)	**Secondary search** (17.10.2024)
**PubMed -** total number of records found	175	10
**PubPsych -** total number of records found	8	2
**CINAHL** - total number of records found	20	1
**EMBASE** - total number of records found	283	23
**Web of Science -** total number of records found	135	17
**EBSCOhost** – total number of records found	12	0
**Total (*after duplicates*)**	633	53
