# Supplementary material for: Prehabilitation for colon cancer surgery: a systematic review of quality-of-life outcomes and a feasibility study
Source: Int J Colorectal Dis. 2026 Jul 25;41(1):165. doi: 10.1007/s00384-026-05203-w (PMC13683530; doi:10.1007/s00384-026-05203-w)
Supplement: Supplementary file 2 [file 384_2026_5203_MOESM2_ESM.pdf]

Page 1

Appendix 2 – Questionnaires included

Note: EQ-5D-5L occurs twice (once at baseline and at post-surgery)

Record ID ______________________________

INITIAL QUESTIONS + Cancer symptoms

Height (in cm) ______________________________

Weight (kg) – Please weigh the patient (Randers patient) or ask the patient (Horsens patient) ______________________________

Have you lost weight within the past 6 months?

If the patient is not aware of the number of kilograms, (please ask them to give their best estimate) ______________________________

How much weight have you gained? If you do not know your exact weight gain, please provide the most accurate estimate possible. ______________________________

If you have lost weight, was it intentional?

- ◯ No
- ◯ Yes

Over the past 2 weeks, how often have you had a reduced appetite?

- ◯ Not at all
- ◯ Several days
- ◯ More than half of the days
- ◯ Nearly every day

Over the past 2 weeks, how often have you felt more tired than usual?

- ◯ Not at all
- ◯ Several days
- ◯ More than half of the days
- ◯ Nearly every day

EQ-5D-5L

PERSONAL CARE	◯ I have no problems washing or dressing myself<br>◯ I have slight problems with washing or dressing myself<br>◯ I have moderate problems washing or dressing myself<br>◯ I have severe problems washing or dressing myself<br>◯ I am unable to wash or dress myself<br><br>(Under each heading, please tick the box that best describes your health TODAY.)
MOBILITY	◯ I have no problems walking around<br>◯ I have slight problems walking around<br>◯ I have moderate problems walking around<br>◯ I have severe problems walking around<br>◯ I am unable to walk around<br><br>(Under each heading, please tick the box that best describes your health TODAY.)
USUAL ACTIVITIES<br><br>(e.g., work, study, housework, family or leisure activities)	◯ I have no problems performing my usual activities<br><br>◯ I have slight problems performing my usual activities<br>◯ I have moderate problems performing my usual activities<br>◯ I have severe problems performing my usual activities<br><br>◯ I am unable to perform my usual activities<br>(e.g., work, study, housework, family or leisure activities)
PAIN / DISCOMFORT	◯ I have no pain or discomfort<br>◯ I have slight pain or discomfort<br>◯ I have moderate pain or discomfort<br>◯ I have severe pain or discomfort<br>◯ I have extreme pain or discomfort<br><br>(Under each heading, please tick the box that best describes your health TODAY.)
ANXIETY / DEPRESSION	◯ I am not anxious or depressed<br>◯ I am slightly anxious or depressed<br>◯ I am moderately anxious or depressed<br>◯ I am very anxious or depressed<br>◯ I am extremely anxious or depressed<br><br>(Under each heading, please tick the box that best describes your health TODAY.)

HEALTH

We would like to know how good or poor your health is TODAY..

This scale is numbered from 0 to 100.
100 epresents the best health you can imagine.
0 represents the worst health you can imagine.

Choose a place on the scale that shows how your health is TODAY.

0 50 100

(Place a mark on the scale above)

HEALTH AND LIFESTYLE

Do you have any of the following diseases?

Tick all boxes that apply to the patient..

☐ No known diseases
☐ COPD
☐ Asthma
☐ Diabetes
☐ High blood pressure (medicated)
☐ Previous heart attack
☐ Kidney disease or poor kidney function
☐ Thyroid disease for which I receive medication
☐ Chronic pain for which I receive stronger pain medication
☐ Other known disease for which I receive regular medication

If other, please specify which disease(s) you receive regular medication for:

____

Do you smoke?

◯ No, I have never smoked
◯ I am a former smoker and have not smoked within the past 8 weeks
◯ I am a former smoker and quit smoking less than 8 weeks agoJa, jeg er
◯ Yes, I am a current smoker

How many alcoholic drinks do you consume per week at the moment?
Indicate the average within the past 4 weeks.

◯ Fewer than 10 drinks per week
◯ 10 or more drinks per week

What was your daily functional level before the surgery?

◯ Fully active without physical limitations
◯ Limited in physically demanding activities, but able to be up and about and manage light housework and office work
◯ Up and about and able to take care of myself, but unable to perform any work. Up and active more than 50% of daytime.
◯ Able to perform necessary self-care, but require rest in bed or seated for more than 50% of daytime
◯ In need of round-the-clock care, unable to manage anything

EQ-5D-5L

PERSONAL CARE

◯ I have no problems washing or dressing myself
◯ I have slight problems with washing or dressing myself
◯ I have moderate problems washing or dressing myself
◯ I have severe problems washing or dressing myself
◯ I am unable to wash or dress myself

(Under each heading, please tick the box that best describes your health TODAY.)

MOBILITY

◯ I have no problems walking around
◯ I have slight problems walking around
◯ I have moderate problems walking around
◯ I have severe problems walking around
◯ I am unable to walk around

(Under each heading, please tick the box that best describes your health TODAY.)

USUAL ACTIVITIES

(e.g., work, study, housework, family or leisure activities)

◯ I have no problems performing my usual activities

◯ I have slight problems performing my usual activities
◯ I have moderate problems performing my usual activities
◯ I have severe problems performing my usual activities

◯ I am unable to perform my usual activities
(e.g., work, study, housework, family or leisure activities)

PAIN / DISCOMFORT

◯ I have no pain or discomfort
◯ I have slight pain or discomfort
◯ I have moderate pain or discomfort
◯ I have severe pain or discomfort
◯ I have extreme pain or discomfort

(Under each heading, please tick the box that best describes your health TODAY.)

ANXIETY / DEPRESSION

◯ I am not anxious or depressed
◯ I am slightly anxious or depressed
◯ I am moderately anxious or depressed
◯ I am very anxious or depressed
◯ I am extremely anxious or depressed

(Under each heading, please tick the box that best describes your health TODAY.)

HEALTH

We would like to know how good or poor your health is TODAY.
This scale is numbered from 0 to 100.
100 epresents the best health you can imagine.
0 represents the worst health you can imagine.

Choose a place on the scale that shows how your health is TODAY.

0 50 100

(Place a mark on the scale above)

QUALITY OF RECOVERY 15

(How have you felt during the past 24 hours? From 0 to 10 Where 0 = at no time [poor] and 10 = all the time [excellent])

Able to breathe without difficulty
(How have you felt during the past 24 hours? 0 to 10, where 0 = at no time [poor] and 10 = all the time [excellent])

○ 0 ○ 1 ○ 2 ○ 3 ○ 4 ○ 5 ○ 6 ○ 7 ○ 8 ○ 9 ○ 10

Able to enjoy food

(How have you felt over the past 24 hours? 0 to 10, where 0 = at no time [poor] and 10 = all the time [excellent])

○ 0 ○ 1 ○ 2 ○ 3 ○ 4 ○ 5 ○ 6 ○ 7 ○ 8 ○ 9 ○ 10

Felt rested
(How have you felt during the past 24 hours? 0 to 10, where 0 = at no time [poor] and 10 = all the time [excellent])

○ 0 ○ 1 ○ 2 ○ 3 ○ 4 ○ 5 ○ 6 ○ 7 ○ 8 ○ 9 ○ 10

Sleept well
(How have you felt during the past 24 hours? 0 to 10, where 0 = at no time [poor] and 10 = all the time [excellent])

○ 0 ○ 1 ○ 2 ○ 3 ○ 4 ○ 5 ○ 6 ○ 7 ○ 8 ○ 9 ○ 10

Able to manage personal care and hygiene independently
(How have you felt during the past 24 hours? 0 to 10, where 0 = at no time [poor] and 10 = all the time [excellent])

○ 0 ○ 1 ○ 2 ○ 3 ○ 4 ○ 5 ○ 6 ○ 7 ○ 8 ○ 9 ○ 10

Able to communicate with friends or family
(How have you felt during the past 24 hours? 0 to 10, where 0 = at no time [poor] and 10 = all the time [excellent])

○ 0 ○ 1 ○ 2 ○ 3 ○ 4 ○ 5 ○ 6 ○ 7 ○ 8 ○ 9 ○ 10

Received the necessary support from doctors and nurses
(How have you felt during the past 24 hours? 0 to 10, where 0 = at no time [poor] and 10 = all the time [excellent])

○ 0 ○ 1 ○ 2 ○ 3 ○ 4 ○ 5 ○ 6 ○ 7 ○ 8 ○ 9 ○ 10

Able to resume work or normal household activities

(How have you felt during the past 24 hours? 0 to 10, where 0 = at no time [poor] and 10 = all the time [excellent])

○ 0 ○ 1 ○ 2 ○ 3 ○ 4 ○ 5 ○ 6 ○ 7 ○ 8 ○ 9 ○ 10

Felt comfortable and in control

(How have you felt during the past 24 hours? 0 to 10, where 0 = at no time [poor] and 10 = all the time [excellent])

○ 0 ○ 1 ○ 2 ○ 3 ○ 4 ○ 5 ○ 6 ○ 7 ○ 8 ○ 9 ○ 10

Had a sense of general well-being
(How have you felt during the past 24 hours? 0 to 10, where 0 = at no time [poor] and 10 = all the time [excellent])

○ 0 ○ 1 ○ 2 ○ 3 ○ 4 ○ 5 ○ 6 ○ 7 ○ 8 ○ 9 ○ 10

Part B

(How have you experienced the following during the past 24 hours? From 0 to 10 Where 10 = at no time [excellent] and 0 = all the time [poor])

Moderate pain
(Have you experienced the following within the past 24 hours? 10 to 0, where 10 = at no time [excellent] and 0 = all the time [poor])

○ 0 ○ 1 ○ 2 ○ 3 ○ 4 ○ 5 ○ 6 ○ 7 ○ 8 ○ 9 ○ 10

Nausea or vomiting
(Have you experienced the following within the past 24 hours? 10 to 0, where 10 = at no time [excellent] and 0 = all the time [poor])

○ 0 ○ 1 ○ 2 ○ 3 ○ 4 ○ 5 ○ 6 ○ 7 ○ 8 ○ 9 ○ 10

Severe pain
(Have you experienced the following within the past 24 hours? 10 to 0, where 10 = at no time [excellent] and 0 = all the time [poor])

○ 0 ○ 1 ○ 2 ○ 3 ○ 4 ○ 5 ○ 6 ○ 7 ○ 8 ○ 9 ○ 10

Felt worried or anxious
(Have you experienced the following within the past 24 hours? 10 to 0, where 10 = at no time [excellent] and 0 = all the time [poor])

○ 0 ○ 1 ○ 2 ○ 3 ○ 4 ○ 5 ○ 6 ○ 7 ○ 8 ○ 9 ○ 10

Felt sad or depressed
(Have you experienced the following within the past 24 hours? 10 to 0, where 10 = at no time [excellent] and 0 = all the time [poor])

○ 0 ○ 1 ○ 2 ○ 3 ○ 4 ○ 5 ○ 6 ○ 7 ○ 8 ○ 9 ○ 10

QUESTION INTERVENTION

(Depending on whether this is case or a control)

Where did the patient undergo colonoscopy/endoscopy prior to surgery?	○ Randers (case)<br>○ Horsens (control)
How many days per week, prior to your surgery, were you physically active for at least 20 minutes (e.g., a walk)?	○ Not at all<br>○ 1 day per week<br>○ 2-3 days per week<br>○ 3-5 days per week<br>○ 5-6 days per week<br>○ Everyday
How many days per week, prior to your surgery, did you on average drink at least 3 protein drinks?	○ Not at all<br>○ 1 day per week<br>○ 2-3 days per week<br>○ 3-5 days per week<br>○ 5-6 days per week<br>○ Everyday<br>○ Other

Have you continued physical activity (at least 20 min.) after the surgery?

◯ Not at all
◯ Yes, to a small extent (1-2 times per week)
◯ Yes, to a some extent (3-4 times per week)
◯ Yes, to a high extent (5-6 times per week)
◯ Yes, to a vey high extent (7 times per week)

Have you continued increased protein intake after the surgery?

◯ Not at all
◯ Yes, to a small extent (1-2 times per week)
◯ Yes, to a some extent (3-4 times per week)
◯ Yes, to a high extent (5-6 times per week)
◯ Yes, to a vey high extent (7 times per week)

Have you changed your eating habits before the surgery?

◯ Not at all
◯ Yes, there are some things I no longer eat
◯ Yes, there is something new I eat that I did not eat before

---

What dietary changes have you made?

____

Have you increased your physical activity level
(by at least 20 minutes per session) before the surgery?

◯ Not at all
◯ Yes, to a small extent (1-2 times per week)
◯ Yes, to a some extent (3-4 times per week)
◯ Yes, to a high extent (5-6 times per week)
◯ Yes, to a vey high extent (7 times per week)

To what extent have you felt able to make a difference in your diagnostic and treatment process before the surgery?

◯ Not at all
◯ To a low extent
◯ To some extent
◯ To a high extent
◯ To a very high extent
